# A Review of LIDAR Radiometric Processing: From *Ad Hoc* Intensity Correction to Rigorous Radiometric Calibration

**DOI:** 10.3390/s151128099

**Published:** 2015-11-06

**Authors:** Alireza G. Kashani, Michael J. Olsen, Christopher E. Parrish, Nicholas Wilson

**Affiliations:** School of Civil and Construction Engineering, Oregon State University, 101 Kearney Hall, Corvallis, OR 97331, USA; E-Mails: michael.olsen@oregonstate.edu (M.J.O.); christopher.parrish@oregonstate.edu (C.E.P.); wilsonn2@oregonstate.edu (N.W.)

**Keywords:** LIDAR, laser scanning, intensity, normalization, correction, calibration, radiometric

## Abstract

In addition to precise 3D coordinates, most light detection and ranging (LIDAR) systems also record “intensity”, loosely defined as the strength of the backscattered echo for each measured point. To date, LIDAR intensity data have proven beneficial in a wide range of applications because they are related to surface parameters, such as reflectance. While numerous procedures have been introduced in the scientific literature, and even commercial software, to enhance the utility of intensity data through a variety of “normalization”, “correction”, or “calibration” techniques, the current situation is complicated by a lack of standardization, as well as confusing, inconsistent use of terminology. In this paper, we first provide an overview of basic principles of LIDAR intensity measurements and applications utilizing intensity information from terrestrial, airborne topographic, and airborne bathymetric LIDAR. Next, we review effective parameters on intensity measurements, basic theory, and current intensity processing methods. We define terminology adopted from the most commonly-used conventions based on a review of current literature. Finally, we identify topics in need of further research. Ultimately, the presented information helps lay the foundation for future standards and specifications for LIDAR radiometric calibration.

## 1. Introduction

Across a wide range of applications, the usefulness of light detection and ranging (LIDAR) data is enhanced by the availability of “intensity” values. To date, LIDAR intensity data have proven beneficial in data registration, feature extraction, classification, surface analysis, segmentation, and object detection and recognition, to name just a few examples. The list of applications also continues to grow rapidly, as LIDAR researchers and practitioners develop new and innovative uses of these data. The primary benefit of LIDAR intensity lies in the fact that it is related to surface reflectance and other surface characteristics. Unfortunately, there are also a number of confounding variables to which intensity is related, including parameters related to the data acquisition geometry, scanning environment, and sensors, themselves. To overcome this issue, a number of techniques have been developed to calibrate, normalize, or otherwise correct the recorded intensity values to produce values that are more useful and more closely related to true surface characteristics.

Despite the rapid progress that has been made, and the wealth of published literature on this topic, there is very little consistency across efforts. Multiple individuals, groups, and organizations are currently applying vastly different processing approaches to LIDAR intensity, and using differing terminology to describe these procedures. Radiometric calibration, intensity normalization, and intensity correction are just a few of the terms used to refer to different processing approaches. The outputs are also given diverse names, including reflectance, albedo, amplitude, normalized intensity, corrected intensity, pseudo-reflectance, and relative reflectance. Even the term “intensity” itself is debated and variously defined. Not surprisingly, researchers, clients, and end users are often confused by these products and the terminology used by data providers to describe them.

In this paper, we seek to address these pressing challenges. Our specific goals are to: (1) provide an overview of basic principles in LIDAR radiometric measurements and data processing; (2) discuss examples of how intensity values are being utilized for representative applications; (3) define consistent terminology (which we accomplish not by inventing new terms or insisting on a purist’s adherence to strict radiometric or photometric usage, but by adopting the most commonly-used conventions based on a review of current literature); (4) lay the foundations for future standards and specifications for LIDAR radiometric calibration; and (5) identify topics in need of further research.

While it is hoped that this paper will prove useful to a broad range of users, the primary intended audience consists of practitioners who want to evaluate different radiometric processing approaches from an implementation perspective and/or LIDAR data consumers who want to better understand (and possibly control, through appropriate contract wording) the types of intensity-derived products that are delivered by LIDAR service providers. Hence, we avoid an elaborate theoretical formulation, while providing implementation-level details and extensive references for interested readers.

## 2. Basics of LIDAR Intensity Measurement

While LIDAR system designs differ markedly between different manufacturers and models, most current systems employ one or more receiver channels using an avalanche photodiode (APD), photomultiplier tube (PMT), or other photodetector to convert the received optical signal to an electrical signal, to which various ranging strategies can be applied. For example, the ranging can be performed in hardware, using a constant fraction discriminator (CFD) and time interval meter, or by digitizing the received signal and applying any of a number of range detection algorithms to the output. Leading edge detection, centroid analysis, and deconvolution are just a few of the methods used to extract individual ranges from the return signal. Many of the photodetectors used in commercial topographic LIDAR systems are designed to be linear, meaning that the output photocurrent is linearly proportional to the input optical power over the detector’s operating range [1].

**Figure 1:**
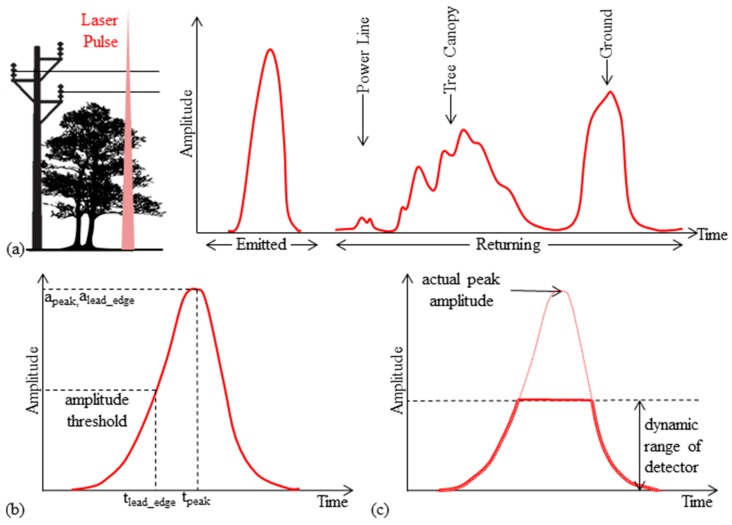
(**a**) Example of the shape of a waveform emitted and returned; (**b**) point selection on the return waveform in the peak detection and leading edge detection methods; (**c**) saturation impact resulting from highly reflective objects close to the scanner, exceeding detection thresholds.

In addition to being used to extract ranges (which can be subsequently georeferenced) through any of the methods listed above, the received signal can be used to extract “intensity” values. While somewhat inconsistent with strict radiometric usage, the term intensity in this context refers to the amplitude of the return signal, which can be the analog electrical signal output from the photodetector or the digitized waveform. Figure 1a shows an example of the shape of a waveform emitted and returned. Usually the peak amplitude is used, but it is important to note that the point(s) selected on the return waveform (analog or digital) for intensity measurement vary from one manufacturer to another and are not necessarily coincident with the points used for range measurement. Figure 1b shows the difference in point selection on the return waveform from the peak detection and leading edge detection methods. For discrete-return systems employing hardware-based ranging and leading-edge detection, another strategy is to delay the intensity measurement by a fixed time after the range measurement. Interested readers are referred to [1] for a detailed discussion.

The amplitude—however and wherever it is measured—is then typically scaled to an 8, 12, or 16 bit dynamic range, at which point it can be provided as an additional parameter in an output LIDAR point cloud, for example using the “intensity” field in the point data records in a LAS file.

**Figure 2:**
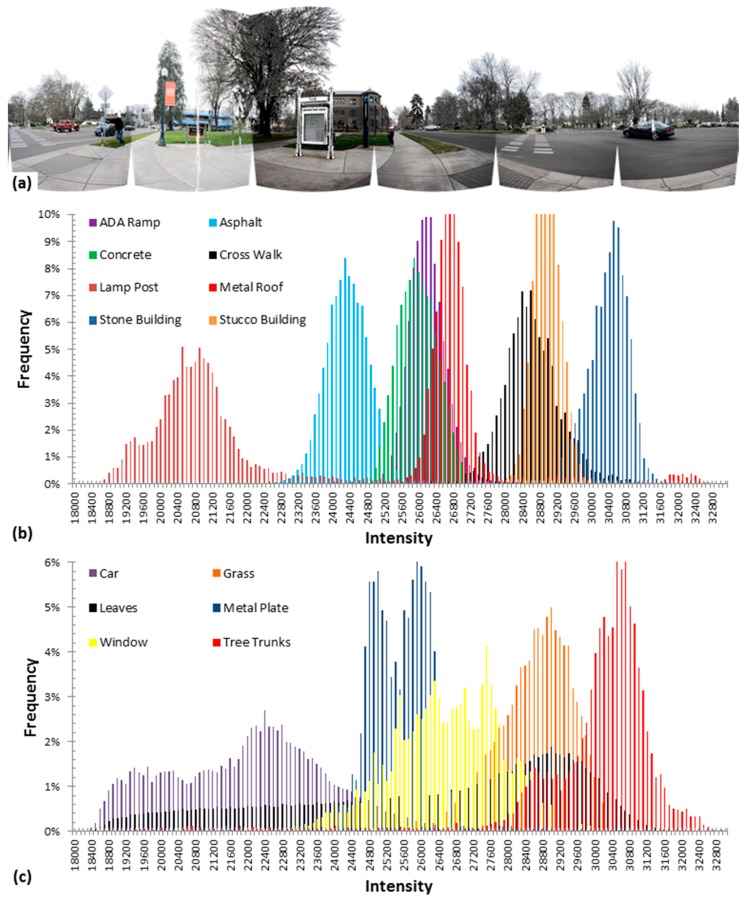
(**a**) Panoramic representation of a scanned scene near an intersection (**b**,**c**) histograms of intensity values measured on different surfaces.

The received optical signal at the detector and, hence, the derived intensity values, are related to the properties of the surface from which the laser pulse was reflected. Therefore, intensity is a potentially useful parameter in that it contains information about the surface. For example, Figure 2 illustrates histograms of intensity values measured by a terrestrial scanner on different surfaces in a street scene. Note that many objects have distinctly different intensity ranges and potentially could be segmented or classified using intensity information.

However, these intensity values (regardless of the specific details of how they are measured and recorded) are also affected by a number of other parameters, including transmittal power, range, angle of incidence, atmospheric transmittance, beam divergence, and detector responsivity (Section 4 will describe these in more detail). For users who wish to use intensity to analyze surface characteristics, these additional system and environmental variables can be considered nuisance parameters. Therefore, processing strategies which aim to remove the effects of these parameters on the intensity data may be desirable to enhance the utility of the data for the user’s intended application. These strategies, the terminology used to describe them, and the characteristics of the output, are the primary focus of the following sections.

## 3. Applications of LIDAR Intensity

The radiometric information provided by scanners has been used alone or as a supplement to other spatial and spectral remote sensing data in a variety of applications. Table 1 presents a summary of some currently-studied applications of LIDAR intensity including remote sensing data registration, land cover classification, natural environment sensing, bathymetry, structural damage detection, and transportation asset management. This list is by no means comprehensive; new applications continue to emerge at a high rate.

**Figure 3:**
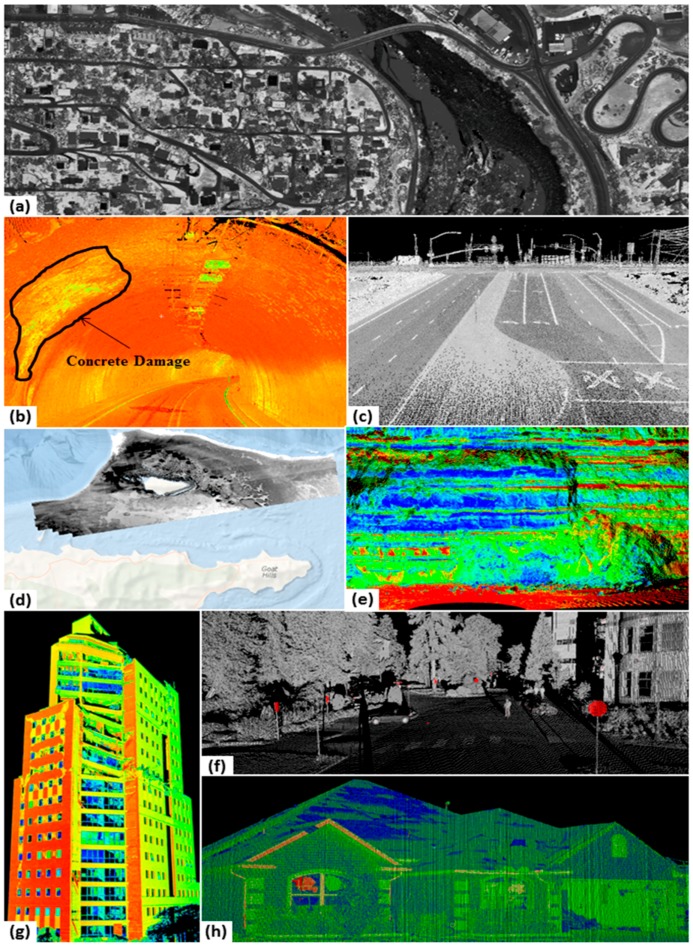
Example applications of LIDAR intensity. (**a**) Intensity image from ALS data; (**b**) intensity shaded point cloud showing damage to concrete in a tunnel (data courtesy of Oregon DOT); (**c**) Intensity shaded point cloud showing pavement lines and striping; (**d**) corrected bottom intensity image for mapping seafloor; (**e**) intensity colored point cloud showing different geologic layers in a cliff; (**f**) detection of reflective signs based on intensity values; (**g**) intensity colored point cloud showing damage to concrete walls after an earthquake; and (**h**) intensity-colored point cloud point cloud showing damage to roof cladding after a tornado.

Figure 3 shows several datasets as examples of applications of LIDAR intensity. Theoretically, materials have different spectral reflectance properties resulting in different backscattering laser intensities. Therefore, the LIDAR intensity can be used as a means to classify and detect different materials in scans of natural or urban environments.

Although some methods involve simply using intensity values to “colorize” the point cloud, another common approach is to generate georeferenced 2D intensity images (Figure 3a). These images can be produced by gridding the data and using different operations such as the (weighted) mean, maximum, or minimum to assign the intensity value of grids containing multiple points. In an extension to 3D, it is also possible to create voxelized representations, in which each voxel stores an intensity value.

A major application of LIDAR intensity that has been widely studied is to classify natural and urban cover surfaces. In initial efforts, Song *et al.* [2] and Charaniya *et al.* [3] indicated that intensity data enables the separation of typical land cover surfaces such as asphalt roads, grass, trees, and house roof captured in ALS scans. Brennan and Webster [4] and Matikainen *et al.* [5] developed methods for detection and classification of building structures. Arnold *et al.* [6] used intensity data to discriminate snow covered areas from bare ice in a glacier. Im *et al.* [7] conducted tests to evaluate different features for land cover classification and found that adding the LIDAR intensity to classification features results in 10% to 20% increase in the accuracy of results. The LIDAR intensity has been also used as a supplement feature with other remote sensing data for land cover classification. Zhou *et al.* [8] used LIDAR intensity data to facilitate land cover classification of shaded areas in aerial images. MacFaden *et al.* [9] used LIDAR intensity for detecting impervious surfaces not detectable in aerial images.

LIDAR intensity is also used to detect common features in multiple sets of remote sensing data for registration. Methods using LIDAR intensity data have been developed for segmentation of multiple scans [10,11,12,13,14] and co-registration of scans and images [15,16,17,18,19,20].

**Table 1:** Example applications utilizing LIDAR intensity information.

Category	Application	References
Cultural Heritage/Virtual Tourism	Analysis of historical paintings/artifacts Digital preservation	[21,22]
Land cover classification	Classification of urban surfaces	[2,3,7]
	Detection and classification of buildings	[4,5]
	Classification of glacier surfaces	[6]
	Supplementing image-based land cover classifications	[8,9]
Remote sensing data registration	Registration of multiple scans by identifying common features	[10,11,12,13,14]
	integration of scans and images by identifying common features	[15,16,17,18,19,20]
Sensing natural environments	Flood modeling and wetland hydrology	[23,24]
	Tree classification, snag detection, and forest understory vegetation cover	[25,26,27,28,29,30]
	Identification of different rock and soil layers	[31]
	Lava flows aging	[32]
	Snow cover change detection	[33]
	Costal land cover mapping	[34]
Bathymetry (using bathymetric LIDAR)	Benthic habitat mapping	[35,36,37,38,39]
	Hydrodynamic and sedimentological properties	[40]
Structural damage detection	Assessment of historic buildings	[41]
	Crack detection of concrete structures	[42,43,44]
	Detection of bridge surface degradation	[45]
	Detection of wind-induced cladding damage	[46,47,48]
Transportation asset management	Detection of road objects and features (e.g., markings, signs, manhole, culverts, *etc.*)	[49,50,51,52,53,54]
	Pavement and tunnel damage detection	[55,56]
	Extraction of road profile	[57]

LIDAR intensity has also proven useful for sensing natural environments. Antonarakis *et al.* [23] and Lang *et al.* [24] developed methods for extracting natural surface roughness information for flood modeling and wetland hydrology. Mazzarini *et al.* [32] and Burton *et al.* [31] used LIDAR intensity data respectively for aging lava flows and sensing rock properties. Several researchers indicated the potential of LIDAR intensity in forest canopy classification and sensing [25,26,27,28,29,30]. LIDAR intensity data was also used for snow cover change detection [33] and coastal land cover mapping [34].

The water penetrating capabilities of bathymetric LIDAR enable the intensity returns to be used in detecting various seafloor features. For example, benthic habitat types can be classified using the relative reflectance values of the returns. Collin *et al.* [35] classified seabed features into four primary categories and used underwater photography to ground-truth the data. Several researchers examined the fusion of bathymetric LIDAR data with hyperspectral data to measure environmental parameters including seafloor reflectance, water depth and water column parameters [36,38,39]. Narayanan *et al.* [37] classified seafloor intensity values into habitat type using decision trees. Figure 3d shows relative reflectance values in which seafloor features can be distinguished. Long *et al.* [40] demonstrates that bathymetric LIDAR intensity waveforms can be used to determine sedimentological and hydrodynamic characteristics. Seafloor reflectance data from bathymetric LIDAR can be even more valuable than its terrestrial/topographic counterparts, due to the challenges typically encountered in obtaining detailed imagery of the seafloor from other airborne or spaceborne remote sensing technologies.

Variations of LIDAR intensity backscattered from intact and damaged materials have enabled advanced structural damage detection and quantification using LIDAR. Armesto-González *et al.* [41] employed LIDAR intensity data to detect degraded stony materials in scans of historic buildings. Several researchers used LIDAR intensity to detect cracks in concrete structural components in their laboratory tests or post-disaster field investigations [42,43,44]. Kashani *et al.* [46,47,48] indicated that the LIDAR intensity data is an appropriate means to automatically detect cladding damage of buildings after wind storm events.

LIDAR intensity data was used directly without any radiometric processing in some early studies [2,3,4], while subsequent work considered the impacts of radiometric calibration and correction. Gatziolis [26] and Korpela *et al.* [27] indicated that correcting the range and intensity data resulted in respectively 9% and 31% improvement in their LIDAR-based canopy classification results. Yan *et al.* [58] demonstrated that applying radiometric correction on scans of an urban area resulted in 9% to 13% improvement in accuracy of their land cover classification. Kaasalainen *et al.* [59] compared the LIDAR intensity data captured from a number of calibration reference targets with their “true” reflectance values obtained by a near-infrared digital camera. The study indicated that the radiometric calibration improves the accuracy of LIDAR-based target reflectance measurements.

## 4. Effective Parameters Influencing Intensity Measurements

**Figure 4:**
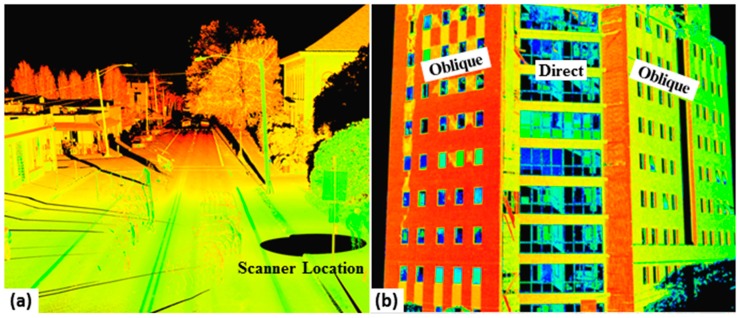

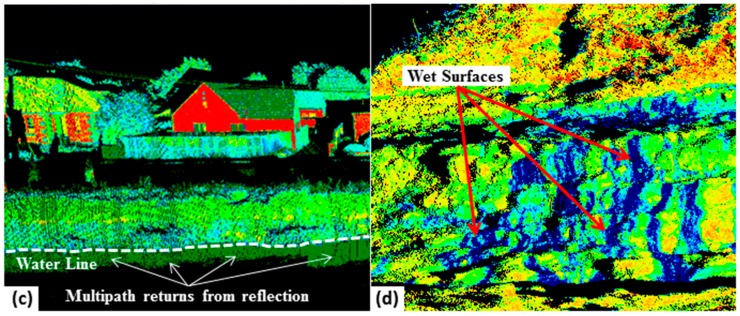
Examples of factors that influence intensity values. (**a**) Degraded intensity values with range on objects such as street lights and asphalt pavement; (**b**) dissimilar intensity values captured on walls with different angles of incidence (larger view in Figure 3g); (**c**) lower intensity values for multipath returns from reflections of the laser off of the water surface; and (**d**) degraded intensity values (blue) due to wet surfaces at a rocky intertidal site.

As mentioned previously, several factors influence LIDAR intensity values that can distort its ability to directly measure reflectance. Table 2 provides a list of effective factors and brief description of their influence. As shown in the first column of Table 2, the effective factors to which intensity values are related can be divided into four main categories of (1) target surface characteristics; (2) data acquisition geometry; (3) instrumental effects; and (4) environmental effects. These factors are discussed in the following subsections. Figure 4 shows examples of variation in intensity values caused by some of these factors.

**Table 2:** Effective factors influencing LIDAR intensity measurements.

Category	Factor	Description	Related References
Target Surface Characteristics	Reflectance (*ρ*)	By definition, surfaces of higher reflectance will reflect a greater portion of the incident laser radiation, thereby increasing the received signal power. In radiometric calibration, this is typically the parameter of interest.	[59,60,61,62,63,64,65]
	Roughness (*ɳ*)	Surface roughness dictates the type of reflection (e.g., specular *vs.* diffuse)	[62,66,67]
Acquisition Geometry	Range (*R*)	The emitted pulse energy decays as a function of range or distance traveled.	[27,58,63,64,65,68,69,70,71,72,73]
	Angle of Incidence (*α*)	Greater angles of incidence typically result in less of the incident laser energy being backscattered in the direction of the receiver, thereby reducing received optical power. Additionally, when the laser beam strikes a surface obliquely, it increases the backscattering cross section.	[58,62,63,64,65,66,68,69,70,71,72]
	Multiple Returns	When a single laser pulse reflects from objects, an attenuation correction can be applied to compensate for the energy split between objects.	[74,75,76]
Instrumental Effects	Transmitted Energy (*E*)	The amount of energy backscattered from targets is related to the amount of energy transmitted with every pulse. Transmitted pulse energy is related to peak transmitted power (which varies with pulse repetition frequency in many systems) and transmit pulse width.	[59,61,65,77]
	Intensity Bit Depth (*-bit) and Scaling	Different scanners use varying bit depth (e.g., 8-bit, 12-bit or 16-bit) when digitizing the return signal. Recorded digital numbers (DNs) are typically scaled to fill the available dynamic range.	[70,78]
	Amplifier for low reflective surfaces	Some scanners amplify the intensity values measured on low reflective surfaces.	[59,60,61,72]
	Automatic gain control (*Ω*)	Some systems (e.g., Leica ALS systems) employ automatic gain control (AGC), which increases the dynamic range that can be accommodated but can also result in discontinuities in the intensity signal, if not compensated.	[27,65,79]
	Brightness reducer for near distances	Some scanners reduce intensity values measured on close objects (e.g., less than 10 m distance).	[21,54,72]
	Aperture Size (*D_r_*)	A larger aperture admits more light, increasing received signal strength.	[60]
Environmental Effects	Atmospheric Transmittance (*T*) or (*η_atm_*)	Radiant energy attenuates in propagating through the atmosphere, as a function of humidity, temperature pressure and other variables.	[58,65,69,70]
	Wetness	Wet surfaces also absorb more energy from the pulse (particularly at the 1.5 micron wavelength used in some systems), resulting in weaker returns.	[61,69]

### 4.1. Target Surface Characteristics

All other parameters being equal, intensity values increase with surface reflectance because a more reflective surface will return more energy from the pulse. An exception is highly reflective surfaces (e.g., mirrors, glass, and water) that can cause specular reflection (mirror effect) and/or multipath. In the case of multipath, range and intensity values are made from pulses reflected from more than one surface and do not represent “true” surface properties [80].

Highly-reflective objects can present some challenges with LIDAR such as saturation and blooming [80]. Figure 1c shows an example of pulse saturation, which occurs with highly reflective objects located close to the scanner. Since the detectors are calibrated to have higher sensitivity to detect weaker returns from less reflective objects such as topography or buildings, returns from these objects exceed the detection threshold, resulting in truncation of the peak of the actual pulse. As a result, the range to the object is often underestimated.

Blooming, in contrast, occurs on highly reflective objects located far from the scanner [80]. These objects appear larger in the point cloud than they actually are because of a bleeding effect of the laser pulse. The laser pulse diverges with distance, resulting in a larger spot size on the object as well as neighboring objects. Hence, the edge of the laser pulse that is not directed at the object, but a neighboring object can partially encompass the highly reflective object. This results in a much higher intensity return on the adjacent object than that would occur if the reflective object were closer.

Reflectance is typically the parameter of interest in radiometric calibration. Reference targets with known reflectance are often used to analyze the impact of material reflectance on LIDAR intensity measurements and for radiometric calibration [59,62,63,64,65,66,72,77,81]. Some researchers also investigated LIDAR intensity values obtained from common natural and artificial materials such as sand, gravel, asphalt, concrete, and brick materials [59,61,62,65,66,72]. More specifics on these methods will be discussed later in the paper in Section 6. In order to extract “true” surface material parameters from intensity, the influence of other effective factors shown in Table 2 need to be eliminated or otherwise reduced.

### 4.2. Data Acquisition Geometry

Factors related to data acquisition geometry such as range (*i.e.*, distance between the sensor and the target) and angle of incidence (*i.e.*, the angle between the emitted laser beam and the target surface normal) greatly influence LIDAR intensity (see Figure 4a,b). The majority of current intensity correction and calibration methods are developed for range and angle of incidence [27,58,62,63,64,65,66,68,69,70,71,72,73]. The primary influence of range on intensity is the fact that the pulse has to pass through more atmosphere and the pulse strength diminishes (*i.e.*, spreading loss). Increases in range and angle of incidence also results in larger target backscattering cross sections. The pulse width increases with range, enlarging the laser footprint and effective backscattering cross section. However, the influence of laser beam divergence on backscattering cross section depends on the shape of targets. In extended targets where the size of target is larger than the laser footprint, the laser beam divergence has greater influence than for point (e.g., a leaf) and linear (e.g., wire) targets where the target area is much smaller than the laser footprint.

The influence of range and angle of incidence varies in ALS and TLS. Ranges are typically much greater and exhibit less percent variability in ALS than in TLS. The scanning range (a function of flying height) in ALS is typically in the range of 600 m to 3000 m, sometimes lower with bathymetric LIDAR. While, except for rare cases of specific time of flight scanners that can capture points up to 6000 m, most TLS systems are typically only capable of measurements less than 300 m in range. TLS data, even those collected within a single scan, contains points with substantially variable ranges and angles of incidence. The TLS data includes much more data at oblique angles, particularly across ground surfaces [57]. Especially in close range scanning, objects such as walls can be found where part of data points have near orthogonal angle of incidence while other parts transition to oblique angles. Additionally in TLS, several scans are often merged and positioned with substantial overlap. This results in an object appearing in one scan at a different angle and range than in another scan, leading to a mix of intensity values on the object in the merged dataset.

In addition to range and angle of incidence, intensity values are affected by how the beam can be split by multiple objects within the path of a single laser pulse, resulting in multiple returns. Attenuation correction processes have been proposed by [74,75] for correcting intensity values of laser returns obtained on forest floors. Reference intensity values are estimated from nearby single peak waveforms that are close to nadir. For simplicity, these points are taken from the same scanline with several screening criteria. The forest floor return intensity values are then adjusted based on an analysis of the integrals of the signals in the waveforms.

### 4.3. Instrumental Effects

Instrumental effects result in different intensity measurements from the same target when different sensors are used. Instrument specific parameters must be known or estimated to develop a LIDAR-based reflectance measurement method that is consistent for different instruments. The aperture size, laser wavelength, beam divergence, and emitted power vary between scanners and can influence the intensity measurement. The aperture size impacts the angular resolution of backscatter measurements [60]. Airborne laser scanners typically have larger aperture sizes (8 < *D_r_* < 15 cm) than terrestrial laser scanners (a few cm) [60]. The laser wavelength often varies in the range of 600 nm to 1550 nm.

The received power is measured, digitized, scaled, and modified by sensors internally; however, this process can vary between different sensors. Discrete waveform scanners may use different peak detection methods causing changes in the range and intensity measurements. Once a returned pulse is detected, the pulse power is digitized to produce intensity which is encoded as an integer number. Typically, the intensity is scaled to a 16-bit value when LAS files are created [78]. Riegl scanners further modify the digitized intensity values and provide two more field values in their LAS exports: amplitude and reflectance, which are explained in [78]. Some scanners further modify intensity measurements, e.g., apply amplifiers for areas with low reflectance or reducers for near-distance areas [72]. Some ALS systems have the ability to adjust the gain using automatic gain control (AGC), which alters the intensity measurements [65]. In addition to the sensor itself, data processing software may apply further scaling or modification influencing intensity measurements. For instance, some software can apply unpredictable intensity scaling to enhance visual appearance [72]. If these internal processing steps are not known, documented, and adjusted with scans, it can be difficult, if not impossible, to calibrate intensity values and measure “true” reflectance values.

### 4.4. Environmental Effects

Atmospheric effects and wetness are the main environmental influences on LIDAR intensity values (except in bathymetric LIDAR, where water column effects dominate). The laser energy is attenuated while passing through atmosphere due to scattering and absorption of the laser photons [58,70]. Small particles suspended in the air such as dust or smoke (aerosol scattering) and air clusters in the atmosphere (Rayleigh scattering) cause laser scattering. Additionally, air molecules such as water vapor, carbon dioxide, oxygen, *etc.* cause laser absorption and energy loss. Atmospheric effects are more influential in ALS than TLS because the laser travels at further ranges as well as vertically with elevation; hence, there are more variance in atmospheric conditions between the scanner and targets.

Another environmental effect is wetness. Kaasalainen *et al.* [61] indicated that moisture can cause a 30% to 50% drop in reflectance of brick samples. For example, Figure 4d shows degraded intensity values (blue) due to wet surfaces at a rocky intertidal site. Intensity values can be used as a filter of erroneous data points. For example, Figure 4c shows lower intensity values on multipath returns that have reflected off of the water surface and onto the cliff before returning to the scanner. Hence, they create a reflection below the water surface that is not representative of the scene. Similarly, intensity information can also be used to filter spurious moisture points within a scan.

### 4.5. Effective Factors in Bathymetric LIDAR 

While the intent of this paper is to focus primarily on topographic LIDAR, we provide a brief treatment of corresponding processing of bathymetric LIDAR to illuminate the similarities and differences between the two. It is also important to note that, due to the difficulty in capturing imagery of the seafloor with conventional imaging techniques, radiometric calibration of bathymetric LIDAR is incredibly important in enabling detailed seafloor characterization.

**Figure 5:**
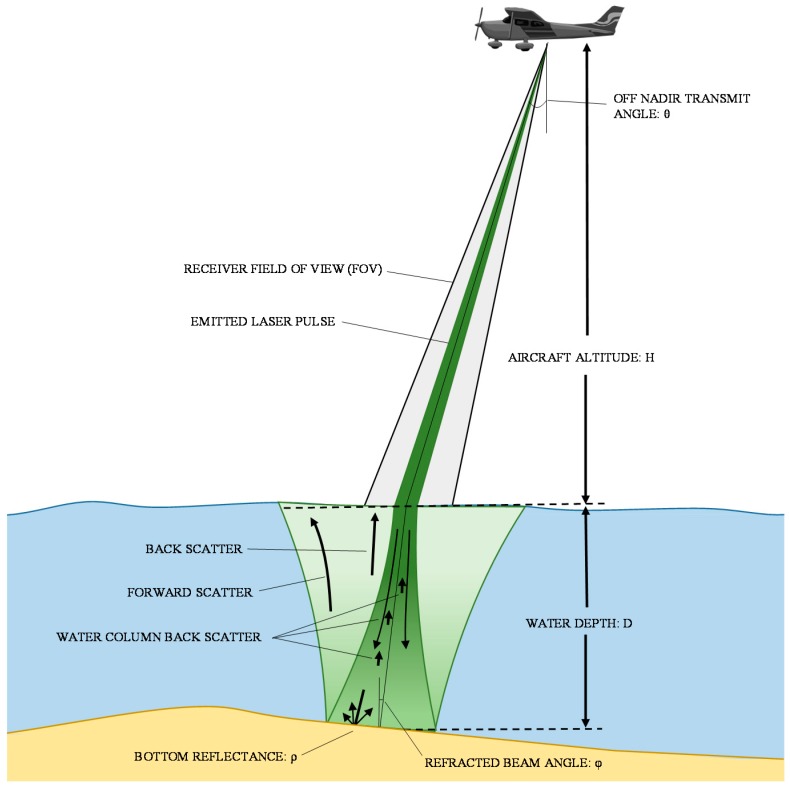
Bathymetric LIDAR acquisition geometry (adapted from [74]).

Working with bathymetric LIDAR intensity requires additional parameters to be considered. The effect of parameters listed in Table 3 on the return intensity value will be demonstrated in Equation (4) (Section 5.2). Acquisition geometry parameters must now consider factors such as the bathymetric angle of incidence, or the angle off nadir at which the pulse is transmitted from the aircraft, aircraft altitude, refracted beam angle, and the receiver field of view [82,83,84,85,86,87]. The water depth has a significant effect on the power of the return pulse, as the power decays exponentially with depth [35,84]. Because depth has such a pronounced effect on intensity values, it is highly important to have accurate depth estimates when calculating the bottom reflectance. The rate at which the return power decays at increasing depth is described by the diffuse attenuation coefficient. This coefficient is defined by [85,86] as the sum of the absorption coefficient and the backward scattering coefficient. For systems with smaller receiver field of view, it is also important to consider a forward scattering coefficient [82,83,84,85,86]. Figure 5 demonstrates typical acquisition geometry.

**Table 3:** Effective factors influencing bathymetric LIDAR intensity measurements.

Category	Factor	Description	Related References
Acquisition Geometry	Water Depth (*D*)	In bathymetric LIDAR, pulse power decays exponentially with the product of water depth and the diffuse attenuation coefficient.	[35,84]
	Off nadir transmit angle (*θ*)	Affects the signal return due to pulse stretching and retro-reflectance of the surface material.	[83,84]
	Receiver field of view loss factor (*F_p_*)	Loss factor due to a receiver FOV is insufficient to accommodate the spreading of the pulse in the water column.	[82,87]
	Aircraft altitude (*H*), refracted beam angle (*Φ*), effective area of receiver optics (*A_r_*)	Other acquisition geometry factors which have an effect on the return power as shown in the bathymetric LIDAR equation (Equation (4)).	[82,85]
	Diffuse Attenuation Coefficient (*K*)	Light traveling through the water column is exponentially attenuated, due to absorption and scattering by particles in the water.	[83,84,86]
	Pulse stretching factor (*n*)	Stretching of the pulse due to acquisition geometry and scattering properties of the water.	[84,85]

## 5. Basic Theory

### 5.1. LIDAR Range Equation

Theoretical or model-driven intensity processing methods are typically based on some form of the LIDAR range equation (also referred to as the laser radar range equation or simply the radar equation), the origins of which lie in the field of microwave radar [88]. This equation relates the received optical power to the transmitted power and other parameters related to the system, acquisition geometry, environment and target characteristics. Numerous forms of the LIDAR range equation can be found in the published literature (e.g., [70,72,88,89,90,91]), but most are equivalent or similar to that given in Equation (1):

(1) $$P_{r}=\frac{P_{t}D_{r}^{2}{\text{η}}_{atm}{\text{η}}_{sys}\text{σ}}{4\text{π}R^{4}{\text{β}}_{t}^{2}}$$

where *P*_r_ = received optical power (watts), *P*_t_ = transmitted power (watts), *D*_r_ = receiver aperture diameter (meters), σ = effective target cross section (square meters)*,* η_atm_ = atmospheric transmission factor (dimensionless), η_sys_ = system transmission factor (dimensionless), *R* = range (meters), and β_t_ = transmit beamwidth (radians). The effective target cross section describes the target characteristics and is given by:

(2) $$\text{σ}=\frac{4\text{π}}{\text{Ω}}\text{ρ}A_{t}$$

where ρ = target reflectance at the laser wavelength (dimensionless), Ω = scattering solid angle (steradians), and *A*_t_ = target area (square meters). Under the assumptions of an extended target (*i.e.*, one that intercepts the entire laser beam) and Lambertian reflectance, a simplified form of the LIDAR range equation can be obtained (e.g., [70]):

(3) $$P_{r}=\frac{P_{t}D_{r}^{2}{\text{η}}_{atm}{\text{η}}_{sys}\text{ρ}}{4R^{2}}\cos{\text{α}}_{i}$$

where α*_i_* = angle of incidence, and all other variables are defined previously (for a discussion on non-Lambertian surfaces, please see [92]). Solving Equation (3) for reflectance, ρ, is mathematically trivial, but, in practice, the challenge lies in obtaining reliable estimates of all other parameters in the equation. Some general approaches include: (1) combining parameters that are unknown but can be assumed constant over a single flight (or, at least, over a single flightline) to create a combined constant; (2) using manufacturer’s system specifications, when available; and (3) using assumed or empirically-determined values. Sometimes, radiometric processing methods that start out with a rigorous theoretical formulation can become more *ad hoc* through the introduction of a number of empirically-determined parameters.

### 5.2. Bathymetric LIDAR Equation

A version of the bathymetric LIDAR equation, adapted from [85], is provided in Equation (4). As with the LIDAR range equation shown above (Equations (1) and (3)), there are also numerous versions of this equation that contain different parameters and are based on different sets of assumptions and simplifications [35,37,38,87,93].

(4) $$P_{r}=\frac{\left(m\right)P_{T}\text{ηρ}F_{p}A_{r}cos^{2}\left(\text{θ}\right)}{\text{π}{\left(n_{w}H+D\right)}^{2}}exp\left(-2\text{n}\left(\text{s},{\text{ω}}_{0},\text{θ}\right)KDsec\left(\phi \right)\right)$$

where, *P*_r_ = received power, *P*_T_ = transmitted power, η = system optical efficiency factor, ρ = reflectance of bottom, *F*_p_ = loss due to insufficient FOV, *A*_r_ = effective area of receiver optics, θ = off nadir transmit angle, *n*_w_ = index of refraction of water, *H* = altitude of LIDAR above water, *D* = bottom depth, *n*(*s*, ω_0_, θ) = pulse stretching factor, *s* = scattering coefficient, ω_0_ = single scattering albedo, *K* = diffuse attenuation coefficient of water, and *ϕ* = nadir angle of LIDAR after entering the water.

The bathymetric LIDAR equation can be used in a similar manner to its topographic-only counterpart in radiometric calibration. However, the situation in bathymetric LIDAR is even more complex, due to the greater number of system and environmental parameters, for which reliable estimates may be difficult to obtain.

## 6. Processing Methods

Raw LIDAR intensity data often undergoes some processing steps to reduce variation caused by the parameters discussed above and sometimes to extract true reflectance information. Unfortunately, there is inconsistency of terminology used in literature to describe these processing steps and procedures. These inconsistencies are further compounded by the fact that LIDAR data has permeated as an important data source for a wide variety of applications and is utilized by with people from diverse backgrounds. Hence, as of yet, there are no standardized definitions for terminology associated with intensity modification methods. Given that there are a wide range of applications supported by intensity information and that significant effort is required to determine and apply modifications, it is not always clear what adjustments have been applied to intensity information in a particular dataset. For the purposes of this paper, we distinguish four levels of intensity processing. Each level increases not only with respect to the accuracy and quality of information but also in effort required:

**Level 0:**  No modification (raw intensity): These are the basic intensity values directly provided by the manufacturer or vendor in their native storage format. They are typically scaled to values of 0–1 (floating point), 0–255 (8-bit integer), or 0–65,535 (16-bit integer), depending on the manufacturer. However, the processes used for scaling the sensor voltages and any adjustments applied are often unknown. Similar results can be obtained for the same scanner model by the same manufacturer; however, there typically is no direct agreement or relationship between values provided by different systems or manufacturers. In this paper, we refer to this as intensity, generically.
**Level 1:**  Intensity correction: In this process an adjustment is made to the intensity values to reduce or ideally eliminate variation caused by one or more effective parameters (e.g., range, angle of incidence, *etc.*). This process is performed by either a theoretical or empirical correction model. Intensity correction ultimately can result in pseudo-reflectance values.
**Level 2:**  Intensity normalization: In this process an intensity image is normalized through scaling to adjust the contrast and/or a shift to adjust the overall “brightness” to improve matching with a neighboring tile or overlapping strip (*i.e.*, a histogram matching or normalization).
**Level 3:**  Rigorous radiometric correction and calibration: In this meticulous process, the intensity values from the LIDAR system are first evaluated on targets with known reflectance, resulting in the determination of calibration constants for the sensor. The calibration constants are then applied to future data that are collected with the system including additional Level 1 intensity corrections to account for any deviations in parameters (e.g., range, angle of incidence). When completed rigorously, this process results in “true” reflectance information. Hence, when radiometric calibration has been applied, consistent data can be obtained from different systems, operated with different parameters settings, and in different conditions. In this paper, we refer to these as reflectance values.

The outputs of Levels 1 and 2 are typically referred to as “relative reflectance” values (sometimes “pseudo-reflectance”), while Level 3 is intended to generate “true” or “absolute” surface reflectance. It is also important to note that the processing levels are not necessarily intended to indicate a particular sequence of processing steps.

Another common approach is to simply apply an *ad hoc* normalization with no proceeding correction. This process is similar to histogram adjustments in image processing software. This workflow is not considered as one of the defined levels, as it is primarily arbitrary and visual in nature so that the intensity values are improved for visual interpretation.

The applications of the different processing levels are application specific and far too numerous to describe in detail. Briefly, however, Levels 1 and 2 are often sufficient for visual analysis and automated land cover classification. On the other hand, combination or comparison of reflectance data acquired with different systems and in different conditions may require a full Level 3 radiometric calibration. Similarly, extraction of true surface albedo requires Level 3 processing. In general, the higher the level the better the results will be for a range of applications. However, lower processing levels can typically be achieved more economically and prove sufficient for a particular user application.

Table 4 and Table 5 summarize some of intensity correction and radiometric calibration methods reported in the literature. The tables are organized by the level of intensity processing and type of scanners used. They also show the types of targets used as well as theoretical and empirical models developed. It should be noted that the tables do not include examples of processing levels 0 and 2. Level 0 is typically completed by the sensor itself. Level 2 processes are not included in the Table and will be summarized later in Section 6.4. The following sections review these methods and the basic theory behind them.

**Table 4:** Selected intensity correction and calibration methods (A, B, C, D denote empirical coefficients, ref denotes a reference).

Reference	Scanner	Level	Targets	Parameters	Theoretical Model	Empirical Model
Luzum *et al.* [94]	(ALS) Optech ALTM 1233	1	n/a	range (R)	$I_{c}=I\times \frac{{R_{i}}^{2}}{{R_{ref}}^{2}}$	n/a
Coren & Sterzai [68]	(ALS) Optech ALTM3033	1	homogenous surface (asphalt road)	range (R)angle of incidence (α) atm. attenuation coeff. (a)	$I_{c}=I\times \frac{{R_{i}}^{2}}{{R_{ref}}^{2}}\times \frac{1}{\cos\text{α}}$	$I_{c}=I\times e^{-AR}$
Starek *et al.* [73]	(ALS) Optech ALTM 1233	1	n/a	range (R)	$I_{c}=I\times \frac{{R_{i}}^{2}}{{R_{ref}}^{2}}\;$	n/a
Hofle & Pfeifer [70]	(ALS) Optech ALTM 3100	1	homogenous surface (asphalt road)	range (R)angle of incidence (α) atm. attenuation coeff. (a) transmitted energy (ET)	$I_{c}=I\times \;\frac{{R_{i}}^{2}}{{R_{ref}}^{2}}\;\times \frac{1}{\cos\text{α}}\times 10^{-2aR}\times \frac{E_{Tref}}{E_{Tj}}\;$	$I_{c}=\;I_{1000}\times f\left(R\right)$ $f\left(R\right)=AR^{2}+BR+\left(1-1000^{2}A\;-1000\;B\right)$
Jutzi and Gross [71]	(ALS) RIEGL LMS—Q560	1	homogenous surface (roof planes)	range (R)angle of incidence (α) atm. attenuation coeff. (a)	n/a	$I_{c}=I\times R^{A}\times e^{2BR}\times cos^{C}\left(\text{α}\right)\times e^{D}$
Korpela *et al.* [27]	(ALS) Optech ALTM3100Leica ALS50	1	homogenous surface	range (R) automatic gain control (Gc)	n/a	$I_{c=\;}I\times \frac{{R_{i}}^{A}}{{R_{ref}}^{A}}+\text{I}\times \text{B}\times \left(\text{C}-\text{Gc}\right)$
Vain *et al.* [95]	(ALS) Leica ALS50-II	1	brightness calibration targets (tarps)	automatic gain control (Gc)	n/a	$I_{c}=A+B\times I+C\times \;I\times \text{Gc }$
Habib *et al.* [96]	(ALS) Leica ALS50	1	n/a	range (R) angle of incidence (α)	$I_{c}=I\times \frac{{R_{i}}^{2}}{{R_{ref}}^{2}}\times \frac{1}{\cos\text{α}}\;$	n/a
Yan *et al.* [58]	(ALS) Leica ALS50	1	n/a	range (R) angle of incidence (α) atm. attenuation coeff. (a)	$I_{c}=I\times \frac{{R_{i}}^{2}}{{R_{ref}}^{2}}\;\times \frac{1}{\cos\text{α}}\times e^{-2aR}$	n/a
Ding *et al.* [69]	(ALS) Leica ALS50-I	1	overlapping scan areas	range (R) angle of incidence (α) atm. attenuation coeff. (a)	$I_{c}=I\times \frac{{R_{i}}^{2}}{{R_{ref}}^{2}}\times \frac{1}{\cos\text{α}}\times 10^{-2aR}$	$I_{c*}=I_{c}\times R^{A}\times 10^{-2BR}\times cos^{C}\left(\alpha \right)\times e^{D}\;$ and Phong model
Ahokas *et al.* [77]	(ALS) Optech ALTM 3100	3	brightness calibration targets (tarps)	range (R) atm. attenuation coeff. (a) transmitted energy (ET) reflectance (ρ)	$I_{c}=I\times \frac{{R_{i}}^{2}}{{R_{ref}}^{2}}\times \frac{E_{Tref}}{E_{Tj}}$	$\text{ρ}=A\times \;I_{c}+B$
Kaasalainen *et al.* [61]	(ALS) Optech ALTM 3100 Topeye MK Leica ALS50	3	sand and gravel	range (R) angle of incidence (α) total atmosphere transmittance (T) pulse energy (E_T_)	method described by Vain *et al.* (2009)	$\text{ρ}=\frac{I_{c}}{I_{ref}}$ where: I_ref_ is reference Intensity measured at the same range of targets
Vain *et al.* [65]	(ALS) Above scanners + Optech ALTM 2033	3	natural & commercial targets, brightness calibration targets (tarps)	range (R)angle of incidence (α) total atmosphere transmittance (T) pulse energy (E_T_)	$I_{c}=I\times \frac{{R_{i}}^{2}}{{R_{ref}}^{2}}\times \frac{1}{\cos\text{α}}\times \frac{1}{T^{2}}\times \frac{E_{Tref}}{E_{Tj}}$	$\text{ρ}=I_{c}\times \frac{{\text{ρ}}_{ref}}{I_{c.ref}}$
Briese *et al.* [97]	(ALS) RIEGL VQ820-G LMS-Q680i VQ-580	3	asphalt road, stone pavement	range (R) angle of incidence (α) detected power (P_r_) empirical calibration constant (C_cal_) reflectance (ρ)	$\text{ρ}=C_{cal}\times \frac{{R_{i}}^{2}}{\cos\text{α}}$	$C_{cal}={\text{ρ}}_{ref}\times \frac{\cos{\text{α}}_{ref}}{{R_{ref}}^{2}}$
Errington *et al.* [98]	(TLS) 3DLS-K2	1	overlapping scan areas	range (R) angle of incidence (α) pseudo-reflectance (ρ)	n/a	The separation model proposed by Pfeifer *et al.* (2008)
Fang *et al.* [21]	(TLS) Z + F Imager5006i	1	White paper targets	range (R) angle of incidence (α) near-distance effect (n(R))	n/a	$I=n\left(R\right)\times \frac{A\times (1-B+B\cos\text{α})}{R^{2}}$
Pfeifer *et al.* [63,64]	(TLS) Riegl LMS-Z420i & Optech ILRIS 3D	3	brightness calibration targets (Spectralon )	range (R) angle of incidence (α) reflectance (ρ)	n/a	(1) $I=g_{1}\left(R\right)\cdot g_{2}\left(\text{ρ}\cos\left(\alpha \right)\right)$ (2) $I=g_{3}\left(\text{ρ}\cos\left(\alpha \right),\;g_{4}\left(R\right)\right)$ where: g1: linear, g2: xA, g3: cubic polynomial, g4: vector valued
Kaasalainen *et al.* [59,60]	(TLS) FARO LS HE80	3	brightness calibration targets (Spectralon)	range (R) reflectance (ρ)	n/a	$\text{ρ}=10^{\frac{\frac{I}{I_{ref}}-A}{B}}$ where: I_ref_ is 99% Spectralon ^®^ reference Intensity measured at the same range of targets
Kaasalainen *et al.* [59]	(TLS) Leica HDS6000	3	brightness calibration targets (Spectralon) gravel	range (R)	n/a	$\text{ρ}=\frac{I}{I_{ref}}$ where: I_ref_ is 99% Spectralon ^®^ reference Intensity measured at the same range of targets

**Table 5:** Selected intensity correction and calibration methods exclusively for bathymetric LIDAR.

Reference	Scanner	Level	Targets	Parameters	Theoretical Model	Empirical Model
Tuell *et al.* [86]	(ALB) Optech SHOALS	3	homogeneous surface (wall covered in painted tiles)	See [86] for derivations of parameters applied.	See Equation (28) in [86] for final model	n/a
Collin *et al.* [35]	(ALB) Optech SHOALS	1	n/a	received power (P_R_) constant combining loss factors (W) transmitted power (P_T_) benthic reflectance (ρ) diffuse attenuation coeff. (K) depth (D)	$P_{R}=W\times P_{T}\times \rho \times e^{-2KD}$	Fourier transform with low-pass filtering, then a nonlinear least squares regression correction for depth.
Wang & Philpot [84]	(ALB) Optech SHOALS	1	n/a	Bathymetric angle of incidence (θ_i_) Derived coefficients (C)	n/a	Correction for bottom reflectance: $f\left(\theta_{i}\right)=C_{1}\times \;\theta_{i}+C_{2}$ Correction for pulse stretching: $g\left(\theta \right)=\{\begin{array}{c} C_{3}\;e^{C_{4}\theta_{i}}\text{, }-90{}^{\circ}<\theta_{i}\leq 0{}^{\circ}\\ C_{5}e^{C_{6}\theta_{i}}\text{, }0{}^{\circ}\leq \theta_{i}\leq 90{}^{\circ} \end{array}$

### 6.1. Theoretical Correction Methods

**Figure 6:**
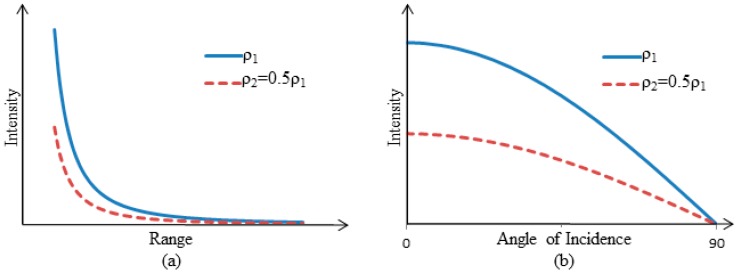
(**a**) Theoretical relationship of intensity measurements *vs*. range shown for two materials with different reflectance (ρ); and (**b**) theoretical relationship of intensity measurements *vs.* angle of incidence shown for two materials with different reflectance (ρ).

Many theoretical corrections have been developed from the LIDAR range equation (Equation (3)). Most theoretical correction methods commonly compensate for variation in intensity data caused by range (R) and angle of incidence (α) [58,65,68,69,70,73,77,88,96]. Based on Equation (3), the received power reflected from extended targets is a function of the inverse range square (Figure 6a) and the cosine of the angle of incidence (Figure 6b). Therefore, in theoretical correction methods, the raw intensity data is multiplied by (R^2^/cos(α)), and then normalized with dividing by a user-defined reference range square (R_ref_^2^) (Equation (5)). The corrected intensity values will be equivalent to the intensity values that would have been measured if the range and angle of incidence for all points were the defined reference range and zero, respectively.

(5) $$I_{c}=I\cdot \;\frac{{R_{i}}^{2}}{{R_{ref}}^{2}}\;\cdot \;\frac{1}{\cos\text{α}}$$

Some limitations should be considered when using the range squared correction. First, the range squared correction works for extended targets but non-extended targets such as leaves and wires with an area smaller than the laser footprint show different range dependency [88]. Based on the LIDAR theory described before, the intensity recorded from non-extended targets is a function of inverse range with higher powers (e.g., 1/R^3^, 1/R^4^). Second, some terrestrial scanners are equipped with brightness reducers for near distances (e.g., less than 10 m) that cause a strong deviation between recorded intensities in near distances and the values calculated by the LIDAR theory equation [21,54,72]. Therefore, the range squared correction is not applicable for near distance intensities (e.g., less than 10 m) recorded by those scanners.

Several approaches to compensate for atmospheric effects have been reported. Generally, the detailed atmospheric conditions and effects are impractical to obtain. However, an approximated value for atmospheric effect should be chosen, which represent the average conditions between the sensor and targets. A common approach is to use radiative transfer simulation models such as MODTRAN. These models can estimate atmospheric transmittance effects based on atmospheric visibility parameters. Vain *et al.* [65] used MODTRAN to estimate the total atmospheric transmittance (T) and corrected intensity values by multiplying with (1/T^2^). Some other studies applied more rigorous models based on the Beer-Lambert Law in which the atmospheric transmittance is a function of range. Höfle and Pfeifer [70] and Ding *et al.* [69] defined an antilog function shown in Equation (6) and Yan *et al.* [58] defined an exponential decay function shown in Equation (7). In both models the attenuation coefficient a must be determined either by simulation models such as MODTRAN or by empirical methods.

(6) $$I_{c}=I\cdot 10^{-\frac{2aR}{1000}}$$

(7) $$I_{c}=I\cdot e^{-2aR}$$

Some correction models account for transmitted energy (E_t_) specifically when the pulse repetition frequency varies or different types of scanners were used during data acquisition [65,70,77]. Correlations between pulse repetition frequency and transmitted pulse energy such as those developed by [89,99] can be used to estimate the transmitted energy in each scan. Intensity values are then divided by the transmitted energy and multiplied to a user-defined reference energy value (Equation (8)). In order to apply this correction, the correlation between pulse repetition frequency and transmitted energy must be known for the scanning sensor used.

(8) $$I_{c}=I\;\cdot \;\frac{E_{Tref}}{E_{Ti}}$$

### 6.2. Empirical Correction Methods

In an empirical correction method, the corrected intensity is defined as a function of one or more variables (e.g., range) and driven from data correlations rather than any physical information or theoretical equation. Empirical methods require homogenous surfaces such as asphalt roads, roof planes, brightness tarps, *etc.*, that are captured in multiple scans with varying settings (e.g., varying ranges). Data captured from these overlapping areas are used to estimate the constant parameters in an empirical correction function, which is then applied to the whole dataset. Empirical methods are suitable when physical and sensor-related parameters in the LIDAR range equation (Equation (3)) are unknown.

Examples of empirical correction methods have been reported in [27,54,68,69,70,71,95,98,100] (see Table 4). Coren and Sterzai [68] used data captured from asphalt roads to estimate the atmospheric attenuation coefficient in an exponential decay correction function as shown in Equation (7). Hofle and Pfeifer [70] adopted an empirical quadratic correction function correlating intensity and range values. Jutzi and Gross [71] and Ding *et al.* [69] devolved empirical intensity correction models including range and angle of incidence. Some studies presented empirical models to compensate for intensity variation caused by automatic gain control systems [27,95].

### 6.3. Bathymetric LIDAR Correction Methods

Corrections for bathymetric LIDAR primarily come in determining systematic and environmental parameters. As with topographic LIDAR, the most rigorous method requires calibrating the system on a surface with known reflectance. Reference [86] offers an example of a rigorous radiometric calibration in which the system is first calibrated against a target, then water optical properties are determined by fitting simulated waveforms to the measured waveform. In [35], the correction approach is a combination of a theoretical approach using a simplified version of the bathymetric LIDAR equation shown above, and an empirical approach using a Fourier Transform with low-pass filtering, then correcting for depth using a nonlinear least squares regression fit of the data. Reference [84] corrects for bottom reflectance using laboratory experiments observations and for pulse stretching using the analytical simulation of [101].

### 6.4. Intensity Normalization Procedures

Following the aforementioned correction procedures, a normalization process is sometimes completed to compensate for differences in overlapping areas between flight-lines or individual scans. This normalization process is also sometimes completed to provide consistency between different sensors.

Qin *et al.* [102] present a normalized reflective factor (NRF), which characterizes the radiometric attributes of a point cloud. In their approach, they apply corrections to intensity values based on energy, geometry and atmospheric effects. A visual analysis of the radiometric attributes is conducted for areas of overlap for quality control purposes. They also utilize hyperspectral imagery to compare the normalized intensity values in specific land cover classes for normalization.

Yan and Shaker [79] propose a sub-histogram matching approach, primarily focused on minimizing the effects of automated gain control. They first perform corrections for geometric and environmental factors. They also provide a slope correction for steep slopes. Next, they identify regions of overlap with wide variability in intensity values. A histogram is generated for each strip. Gaussian components were then fit for sub-histograms found within the histogram. The intersections of these individual Gaussian components were then used as match strips between each strip. The process is then repeated for all overlapping strips and histogram equalization techniques were then applied such that the data were consistent between the strips.

Teo and Yu [54] propose a normalization approach that considers adjacent strips as well as multiple scanners in a mobile laser scanner. In their approach, they employ an empirically-derived piecewise polynomial function for the range correction to account for close-range effects (e.g., <10 m). They then compare the maximum and minimum amplitude differences between strips for the intensity normalization to compute the normalization adjustment. The improvement is evaluated by comparing the mean amplitude differences before and after correction.

### 6.5. Radiometric Calibration with Reference Targets

Reference targets with known reflectance values are required for extracting true reflectance values from intensity data. Brightness reflectance targets such as tarps and Spectralon^®^ targets with known nominal reflectance have been used in several studies [59,60,63,64,65,77,95]. Some studies performed laboratory or *in situ* measurements to determine reflectance values for available natural and commercial objects such as sand, gravel, asphalt, concrete, and brick materials and then used them as reflectance calibration targets [61,62,65,97].

Two main calibration procedures were reported in literature. A common procedure is to separate the correction and calibration steps [61,65,69,77,97]. In this approach, first a theoretical or empirical correction model (explained in Section 6.1 and Section 6.2) is applied to reduce variations in intensity data. Next corrected intensity values are converted to reflectance values by using empirical correlation functions driven from data captured on calibration targets. Another approach [59,60,63,64] combines the correction and calibration steps.

## 7. Summary of Challenges and Future Direction

As LIDAR intensity information continues to become increasingly useful in a wider range of applications with a diverse audience, greater emphasis is being placed on correction and calibration processes. A significant amount of insightful work has been completed to-date, in order to improve the utility of these intensity values. However, as of yet, there is no standard approach for correction or calibration implemented across manufacturers. In some cases, this is further compounded with no consistency between units from different systems from the same manufacturer. Numerical scaling and units to represent intensity are also not consistent between manufacturers of both hardware and software. While relative differences between intensity values tend to be generally consistent for a particular system and scan, they may be vastly different across different systems, scans, acquisition geometries, *etc*. This creates challenges when using or developing filtering or classification algorithms based on intensity values.

While a significant amount of research has been conducted on correction or calibration methods, the selection and usage of parameters, as well as simplifying assumptions in the models, are still widely inconsistent between studies. In particular, no methods were found that can consider the influence of some key parameters (e.g., pulses with multiple returns will have lower intensity values since the beam footprint is spread across multiple objects).

While formulating this review of current literature, several knowledge gaps were identified, including the need to:

1. Develop relationships and unifying research for consistent intensity values/measures between LIDAR systems designed for platforms such as airborne, mobile, and terrestrial. Currently much research between these systems remains distinct; however, there are many similarities between these systems.
2. Evaluate and account for the influences of surface characteristics such as roughness or wetness.
3. Clarify what level of intensity processing is needed (or useful) for specific applications. For some applications, a Level 0 intensity value may prove sufficient. However, for advanced classifications (e.g., determination of plant species), Level 3 calibration may be required.
4. Variance of intensity across wavelengths. The wavelength of LIDAR systems can also vary significantly. Even if a “true” reflectance is calculated from the intensity values, it is important to consider that such a reflectance only applies at the specific wavelength of the system. Many of the parameters described in this review are a function of the wavelength used. Hence, we recommend for future studies that the wavelength be included as a subscript of presented reflectance values (e.g., ρ_532_) obtained via LIDAR.

In addition to future research efforts, we provide the following recommendations to future data exchange standard formats such as the ASPRS “LAS” or the ASTM “E57” formats to better communicate processing completed with intensity data from a LIDAR scan. First, the addition of an attribute in the header could indicate the level of intensity processing applied. Second, an attribute field for the wavelength of the system would be helpful to provide context to these intensity values. Additionally, for critical applications both corrected and original values could be stored. Finally, a detailed description of the process should be documented in the metadata.

As calibration methods continue to evolve, it is likely that future LIDAR systems will be capable of directly providing reflectance values on-board the hardware. Software solutions will continue to utilize this information further, improving processing workflows for a wide range of applications. The improved sensitivity will also result in LIDAR being utilized for a new host of applications that have not been envisioned.
